# Unveiling the underlying complexities in breeding for disease resistance in crop plants: review

**DOI:** 10.3389/fpls.2025.1559751

**Published:** 2025-07-15

**Authors:** Rutuparna Pati, Surinder Sandhu, Ankita K. Kawadiwale, Gagandeep Kaur

**Affiliations:** Department of Plant Breeding and Genetics, Punjab Agricultural University, Ludhiana, India

**Keywords:** pathogen, pathogenesis, plant breeding, plant disease, resistance, Flor’s hypothesis, RLP/RLK, plant immunity

## Abstract

Biotic stress significantly contributes to global crop losses, posing a major threat to food security and agricultural sustainability. While conventional plant breeding techniques have successfully enhanced crop resistance to pathogens, the perpetual emergence of new pathogens and the need to develop varieties with effective, stable, and broad-spectrum resistance in the shortest feasible time remain formidable challenges. The rapid delivery of these technologies to stakeholders further underscores the urgency for innovative approaches. This review delves into the complexities of breeding for disease resistance in crop plants, tracing its historical evolution and highlighting recent advancements in genetic and genomic technologies. These advancements have significantly deepened our understanding of host-pathogen interactions, enabling the identification of key genes and mechanisms governing resistance. We aim to offer insights into how historical perspectives and cutting-edge innovations can guide breeders in designing robust resistance strategies. Ultimately, this work seeks to empower breeders with actionable knowledge and tools to address the dynamic challenges posed by pathogens, paving the way for a more resilient and adaptable agricultural landscape.

## Introduction

The yield loss estimates due to biotic stresses like pathogens at a global level for major cereal crops are- wheat (21.5%), rice (30.0%), maize (22.5%), potato (17.2%) and soybean (21.4%) (Savary et al., 2019). All crop plants are affected by diseases caused by fungal, bacterial, nematode and viral pathogens, leading to significant yield losses Throughout history, plant disease epidemics have inflicted catastrophic losses, crippling food production and triggering severe social and political upheavals (Etherton et al., 2024). The Irish potato famine of 1845 remains a stark reminder of the devastation caused by unchecked pathogens (Ristaino et al., 2021). More recently, the resurgence of Panama disease in bananas, caused by *Fusarium oxysporum* f. sp. *cubense* TR4, underscores the relentless and evolving nature of these threats, posing a continuous challenge to global food security (Oraon et al., 2024). Similarly, each year, around the globe, about 20% of wheat yield is lost due to some of the major diseases such as rusts, smut, viral, nematode and bacterial diseases causing great economic loss (Singh et al., 2023). These ever-emerging diseases threaten to destabilize the strong crop production systems, which ultimately leads to potential food shortages and increased food prices (Oraon et al., 2024). Preventing the invasion and spread of pathogens and pests is, therefore, critical to sustaining crop yield and quality. Plant breeders aim to deploy diverse resistance sources to achieve durable disease control. However, pathogen populations, as well as host populations, are highly dynamic and constantly evolving to produce new races. Encounters of hosts and pathogens result in so-called ‘arms races’, whereby hosts evolve resistance to pathogens while pathogens strive to develop counter-measures to evade host surveillance and to achieve a successful infection (Sironi et al., 2015). Hence, a successful resistance breeding program necessitates a deeper understanding of genetic diversity for resistance, continuous evaluation of pathogen evolutionary dynamics and the integration of traditional breeding practices with modern technologies. This review explores historical perspectives, the genetic basis of resistance breeding and the critical role of conventional strategies, alongside cutting-edge tools and technologies, in unravelling host-pathogen interactions and mitigating potential yield losses.

## Historical perspectives and evolution of disease resistance

**Figure 1:**
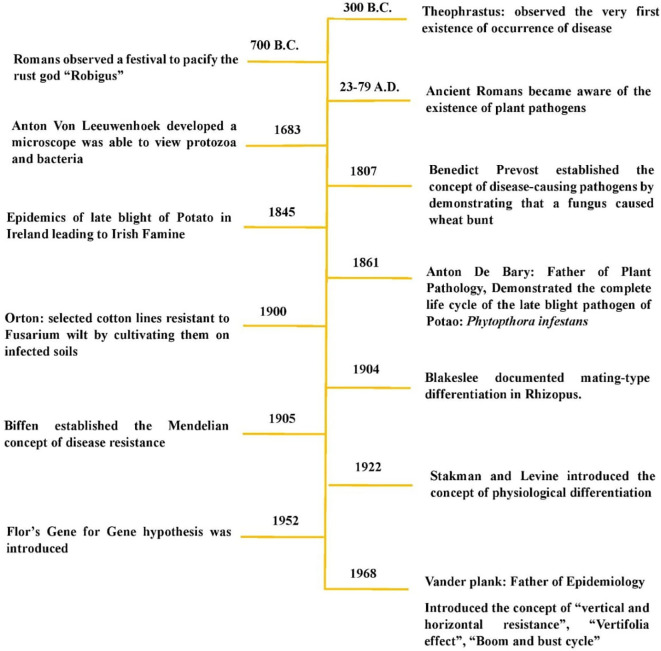
Milestones in genetics of disease resistance.

The first attempt to understand the phenomenon of disease resistance was made by Theophrastus in the third century B.C., when he observed that different cultivated plant varieties exhibited varying capacities to avoid diseases. Ancient Greeks, such as Theophrastus (347–288 B.C.), and ancient Romans, such as Pliny (23–79 A.D.), were well aware of plant diseases (Agrios, 1988; Fry, 1982). Theophrastus, the Father of Botany, described scorch, rot, scab, and blight in his Historia Plantarum (Chester, 1948; Keitt, 1959). He noted the occurrence of certain diseases in specific plants and observed that rust on cereals was often worse in low areas than on high ground (Horsfall and Cowling, 1977; Keitt, 1959). The early Romans attempted to pacify their Gods of Rust, Robigo (male) and Robigus (female), as early as 700 B.C. with an annual festival called Robigalia (Horsfall and Cowling, 1977; Stakman, 1958). Later, Benedict Prevost established the concept of disease-causing pathogens by demonstrating that wheat bunt was caused by a fungus (Prévost, 1807). The formal study of plant pathology advanced in the 19th century when Heinrich Anton de Bary provided experimental evidence that specific fungi caused plant diseases, refuting the theory of spontaneous generation (de Bary, 1884; Tronsmo et al., 2020). In 1904, Blakeslee documented mating-type differentiation in Rhizopus (Blakeslee, 1904a and b). The following year, one of the major successes in the field of disease resistance was achieved by Biffen, who showed that resistance to yellow rust (*Puccinia striiformis*) in wheat was governed by a recessive gene, segregating in a 3:1 ratio in the F_2_ generation. He established the Mendelian concept of disease resistance (**Figure 1**).

However, breeding for disease resistance is thought to have originated with Orton’s work in 1900, when he selected cotton lines resistant to Fusarium wilt by cultivating them on infected soils (Jones, 1931). Earlier, in 1894, Erikson proposed that pathogens with morphological similarities differed in their ability to attack related host species (Ainsworth, 1981). Barrus in 1911 later expanded on this concept , demonstrating that isolates of the bean anthracnose fungus (*Colletotrichum lindemuthianum*) varied in their ability to infect different bean isolates, ultimately leading to the concept of physiological races or pathotypes (Barrus, 1911). In 1940, Johnson and Newton further refined the concept of pathogenicity, demonstrating that a pathogen’s ability to infect a host is genetically determined (Johnson and Newton, 1940). Consequently, both host resistance and pathogen infectivity are governed by genetic factors. The concept of physiological differentiation, introduced by Stakman and Levine in 1922, eventually led to Flor’s gene-for-gene hypothesis, formally proposed in 1951. This hypothesis established that host resistance and pathogen virulence interact in a genetically reciprocal manner - an idea that remains widely accepted and actively discussed today.

## Gene for gene hypothesis: Flor’s enduring legacy

The very first existence of underlying genetics governing disease resistance in plants was established by R. Biffen’s work on wheat genetics (Biffen, 1905), along with the concept of physiological races by Stakman and Levine in 1922 (Stakman and Levine, 1922), which proved to be a stepping stone in the validation of Mendel’s laws of genetics. However, the accurate relationship between these hosts and pathogen-associated mechanisms was still unknown (Dodds, 2023). The first extensive seminal work on the genetic interaction between flax and its obligate biotrophic rust pathogen, *Melampsora lini*, in the 1940s provided significant insights into disease resistance in plants. The gene-for-gene relationship was first explicitly described in Flor’s 1942 paper, Inheritance of Pathogenicity in *Melampsora lini* (Flor, 1942). The fact that the sexual cycle of *M. lini* occurs on flax rather than an alternate host made Flor’s crossing experiments more practical and successful (Dodds, 2023). According to Flor’s original statement (1942), the gene-for-gene hypothesis states that “plants contain a single dominant resistance (R) gene to specifically recognize pathogens containing complementary avirulence (*Avr*) genes, giving rise to resistance and avirulence inherited dominantly, whereas susceptibility and virulence are inherited recessively. Any loss or alteration of the respective gene by either partner does not prevent disease development” (Flor, 1942). On the varieties of flax that have one gene for resistance to the avirulent parent race, F_2_ cultures segregate into mono-factorial ratios (**Table 1**). Some other flax rust races showed differential responses on these lines, and Flor (1946) interpreted that either different Avr genes were located in very close proximity or that different allelic variants of a single Avr locus showed different patterns of recognition with the corresponding set of host R genes. Thus, the ‘gene-for-gene’ model was never intended to mean one way explanation for one-to-one relationship between bi-allelic loci as has been interpreted many times since Flor’s data showed otherwise. He has explicitly described more complex specificity relationships between allelic loci in both the host and pathogen. On varieties having 2,3, or 4 genes for resistance to the avirulent parent race, the F_2_ generation segregates into bi-, tri-, or tetra-factorial ratios (Flor, 1971) (**Table 2**).

**Table 1:** Gene combinations and disease reactions.

Pathogen Genotype	Host Genotype	
	R1	r1
**AVR1**	**-**	**+**
**avr1**	**+**	**+**

- Incompatible Reaction + Compatible reaction.

Source: Agrios (2005) R1: Dominant resistant gene, r1: Recessive , susceptible gene; AVR1: Avirulence dominant gene, avr1: Virulence gene.

**Table 2:** Complementary Interaction of two host genes for resistant (R1 and R2 loci) and the corresponding two pathogens (A1 and A2) for virulence.

Pathogen Genotype	Host Genotype			
	R1R2	R1r2	r1R2	r1r2
**A1 A2**	**-**	**-**	**-**	**+**
**A1a2**	**-**	**-**	**+**	**+**
**a1A2**	**-**	**+**	**-**	**+**
**a1a2**	**+**	**+**	**+**	**+**

Source: Agrios (2005) - Resistant; + Susceptible.

**Figure 2:**
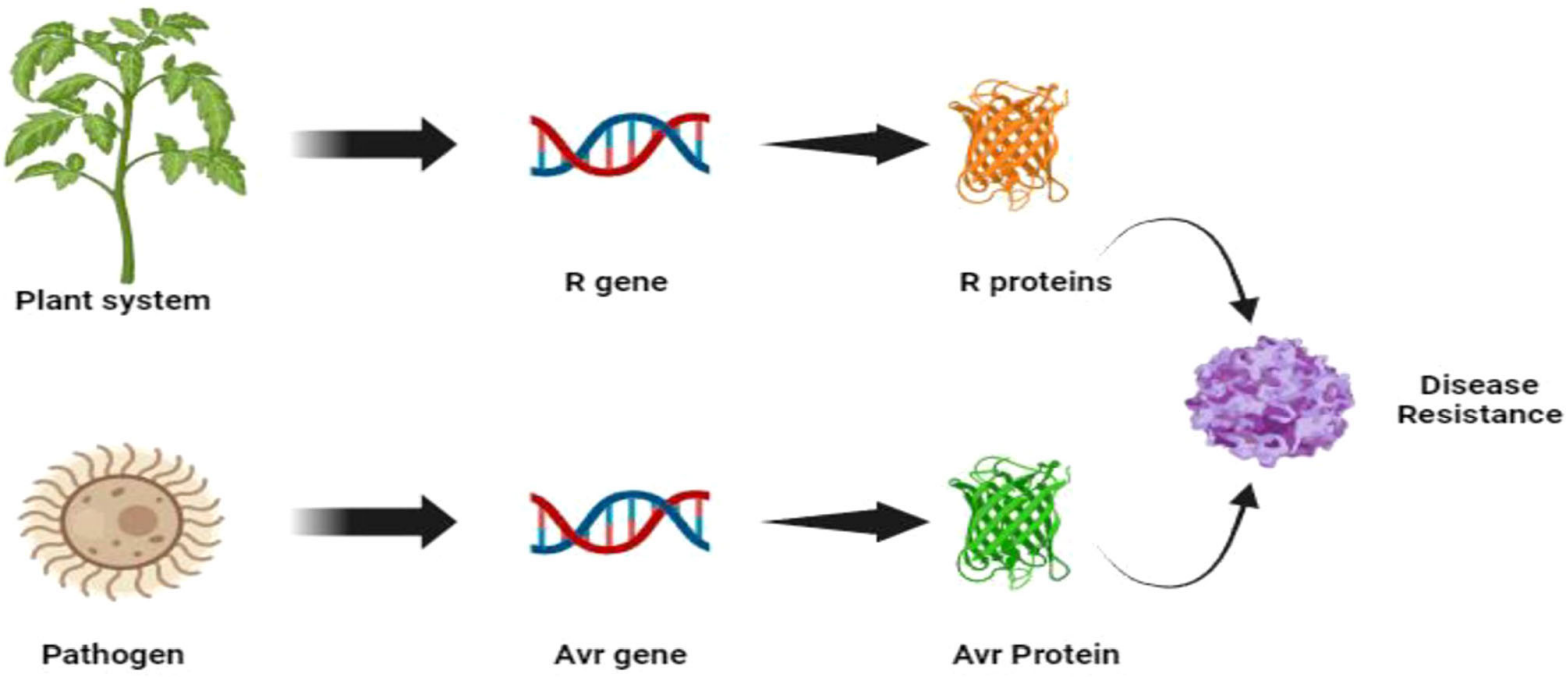
Plant pathogen interaction and development of disease resistance mechanisms (Flor’s hypothesis).

Modern molecular insights into Flor’s work also further revealed that the resistance reaction comes from a complex network of interactions between an array of effector molecules secreted by a pathogen, which is recognised by specific receptor molecules secreted by the plant host (**Figure 2**). However, when the plant loses its ability to recognize the avirulence protein or the effectors molecules from pathogens, its resistance to biotrophic diseases is lost or altered (Dodds, 2023). The first identification of Avr genes from bacterial pathogens in the 1980s (Staskawicz et al., 1984), along with the subsequent realization that these genes encode effector proteins delivered into host cells via the bacterial type III secretion system (Kvitko and Collmer, 2023), provided key insights into host-pathogen interactions. However, Flor’s theory was initially proposed in the context of biotrophic infections. Necrotrophic pathogens differ from biotrophic pathogens, like rusts and mildew, in that they require dead or dying cells to acquire nutrients. This kind of pathogen acts in two manners, i.e., compatible and incompatible reactions (**Tables 3A**, **3B**). Recognition of a fungal necrotrophic pathogen effector by the product of a dominant host gene leads to a compatible interaction (disease susceptibility), and the lack of recognition of the pathogen leads to resistance. Therefore, in plant-necrotroph interactions, plant genes that actively recognise the pathogen are considered susceptibility genes as opposed to plant-biotroph interactions, where they act as resistance genes that proliferate. Though this is an oversimplification of the phenomenon, it laid the foundation for understanding the plant-pathogen interaction (Singh, 2016).

**Table 3a:** Gene for gene model systems for biotrophic pathogens.

Resistance Gene	Virulence Gene	
(Biotrophic pathogens)		
	Avr1	avr 1
R1	Resistant	Susceptible
r1	Susceptible	Susceptible

**Table 3b:** Inverse gene for gene model systems for necrotrophic pathogens.

Resistance Gene	Virulence Gene	
(Necrotrophic pathogens)		
	NE	ne
S	Susceptible	Resistant
s	Resistant	Resistant

Different plant–pathogen interaction mechanisms. (A) Gene-for-gene model, adapted from Flor (1971). (B) Inverse gene-for-gene model, adapted from Friesen et al. (2007). R, resistant gene; r, absence of resistant gene; Avr, avirulence gene; avr, absence of avirulence gene; NE, necrotrophic effector; ne, absence of necrotrophic effector; S, susceptibility gene; s, absence of susceptibility gene.

## Types of resistance

### Qualitative disease resistance

**Figure 3:**
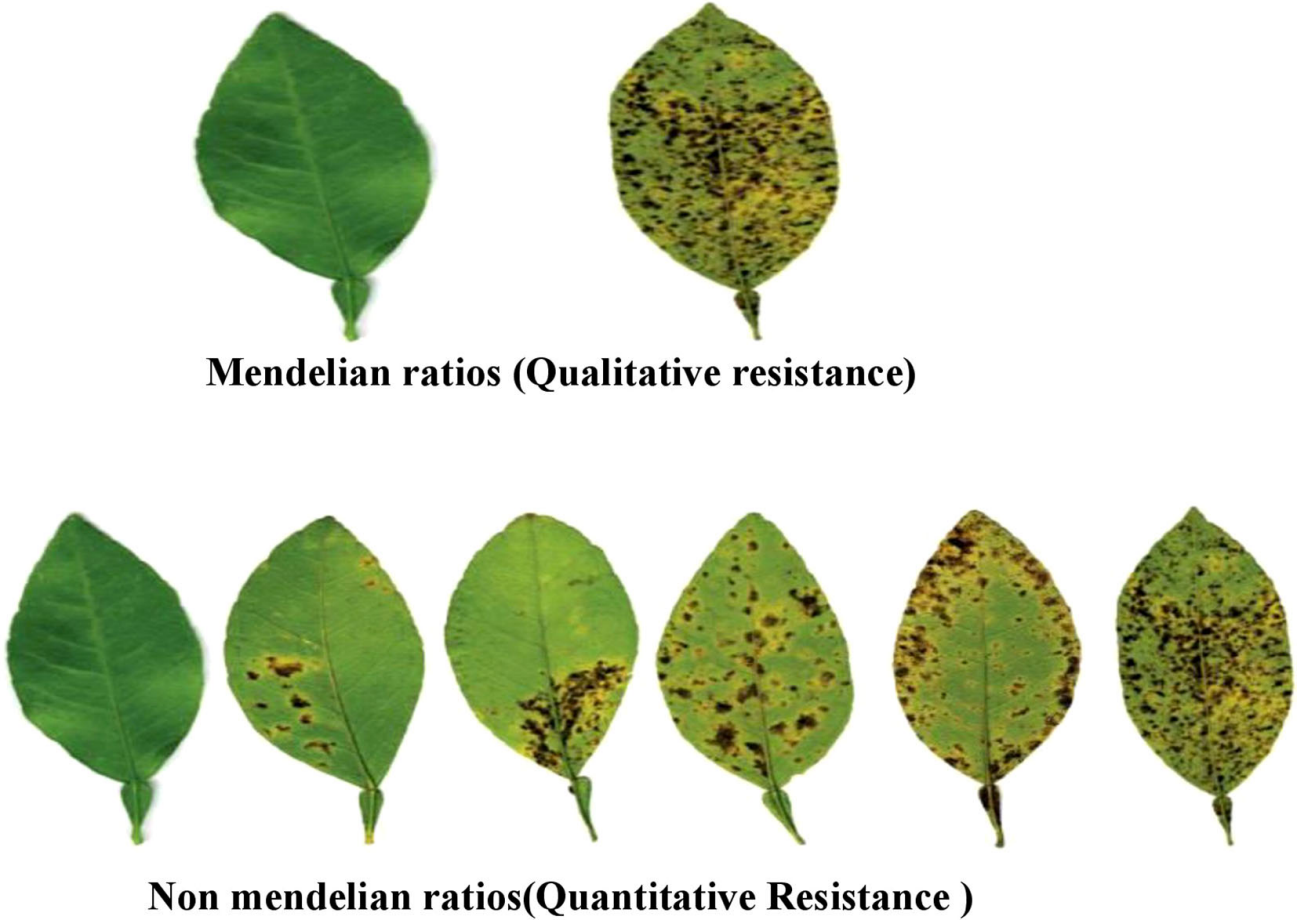
Variable phenotypic expression observed in qualitative and quantitative resistance reaction.

Qualitative disease resistance in plants is primarily governed by a single resistance (R) gene, which provides specific and often complete protection against particular pathogens. This type of resistance is typically race-specific, conferring immunity to certain pathogen strains but remaining vulnerable to pathogen evolution. Studies on qualitative resistance have greatly advanced our understanding of pathogen recognition and host response mechanisms (Jones and Dangl, 2006). Major resistance genes, though not exclusively, encode proteins involved in pathogen recognition. While R genes predominantly exhibit dominant phenotypes, recessive resistance genes also exist (Santa-Cruz et al., 2014; Denby et al., 2004; Jamann et al., 2014). With the advent of advanced genome editing technologies, identifying and cloning R genes has become more efficient, enabling precise modifications to enhance their functionality and durability. Despite these advancements, the longevity of qualitative resistance remains a significant challenge, as pathogens rapidly evolve to circumvent single-gene resistance. Therefore, integrating qualitative resistance with quantitative resistance-mediated by multiple genes providing partial and broad-spectrum protection—is considered a more effective strategy for sustainable disease management in crops (Gou et al., 2023) (**Table 4**; **Figure 3**).

**Table 4:** Comparison between qualitative and quantitative resistance.

Feature	Qualitative Resistance	Quantitative Resistance
Pathotype specificity	Specific	Non-Specific
Nature of gene action	Oligogenic	Polygenic
Response to pathogen	Hypersensitive	Resistance response
Selection and evaluation	Relatively easy	Relatively difficult
Host-Pathogen Interaction	Present	Indirect interactions involving complex of genes and triggering of specific defence mechanisms (Corwin and Kliebenstein, 2017)
Efficiency	Effective against specific race	Variable but operate against all races

### Quantitative disease resistance

QDR is defined by a partial and durable resistance effect, which is generally pathogen race-nonspecific but species-specific (Wisser et al., 2005; Poland et al., 2009; Kliebenstein and Rowe, 2009). This form of resistance is often the primary, if not the only, defence against necrotrophic pathogens and even some biotrophic pathogens, such as *Xanthomonas oryzae* pv. *oryzicola*, the causal agent of rice bacterial streak (Kliebenstein and Rowe, 2009; Hu et al., 2009). One possible source of variation in quantitative resistance phenotypes is the differential expression of genes contributing to the partial resistance effect observed for each gene. Genome-wide studies have identified a large number of plant defence-related genes within quantitative trait loci (QTL) regions, highlighting their role in quantitative resistance (Wisser et al., 2005).

Unlike qualitative resistance, which relies on single resistance (R) genes and is often race-specific, QDR is more durable due to its polygenic nature, making it less susceptible to pathogen adaptation and evolution. Gou et al. (2023) emphasized the importance of integrating QDR traits into breeding programs to develop sustainable and long-lasting resistance crops. This strategy strengthens ongoing efforts to enhance crop resilience while reducing dependence on chemical controls. However, as with most quantitative traits, unravelling the genetic basis of QDR remains challenging, as the relationship between phenotypes and underlying molecular mechanisms is not yet fully understood.

## Plant immune responses: qualitative and quantitative resistance mechanism

Unlike mammals, plants lack adaptive immunity but possess an innate immune system in each cell, with systemic signalling capabilities from infection sites (Jones and Dangl, 2006). In 2006, Jones and Dangl proposed a coevolutionary model to explain modern concepts of plant-pathogen interactions, known as the ‘zigzag’ model, which describes two branches of the plant immune system. The first branch detects conserved microbial molecules, known as pathogen‐associated molecular patterns (PAMPs) or microbe‐associated molecular patterns (MAMPs), and activates a defence response termed pattern‐triggered immunity (PTI). The second branch, effector‐triggered immunity (ETI), responds to virulence factors called effectors, which suppress PTI. As an extension of Harold Flor’s classical “gene-for-gene” theory, the zigzag model integrates multi-level plant immunity responses of varying specificity and amplitude while emphasizing the continuous evolutionary adaptation of both plants and pathogens in their interactions (Shafikova and Omelichkina, 2011).

Upon infestation, pathogens produce elicitors known as pathogen- or microbe-associated molecular patterns (PAMPs/MAMPs), including peptides, metabolites, cell wall-degrading enzymes, and toxins, to suppress primary plant defence (Boller and Felix, 2009; Dodds and Rathjen, 2010; Giraldo and Valent, 2013; Wirthmueller et al., 2013). In response, the damaged host generates damage-associated molecular patterns (DAMPs), such as plant signalling molecules (Boller and Felix, 2009). These elicitors (PAMPs/MAMPs/DAMPs) are recognized by pattern recognition receptors (PRRs), which are synthesized in the endoplasmic reticulum and transported to the plasma membrane (Frescatada-Rosa et al., 2015). Pattern recognition receptors (PRRs) on the cell membrane identify pathogen-associated molecular patterns (PAMPs), while wall-associated kinases (WAKs) recognize damage-associated molecular patterns (DAMPs) resulting from cellular damage during infection. As the first line of defence, PAMP/MAMP recognition triggers downstream gene activation, leading to either symptomless resistance or a race-non-specific hypersensitive response, collectively known as PAMP-triggered immunity (PTI) or non-host resistance (Baxter et al., 2014; Boller and Felix, 2009; Dodds and Rathjen, 2010; Macho and Zipfel, 2015; Stael et al., 2015; Trda et al., 2015; Uma et al., 2011; Waszczak et al., 2015).

**Figure 4:**
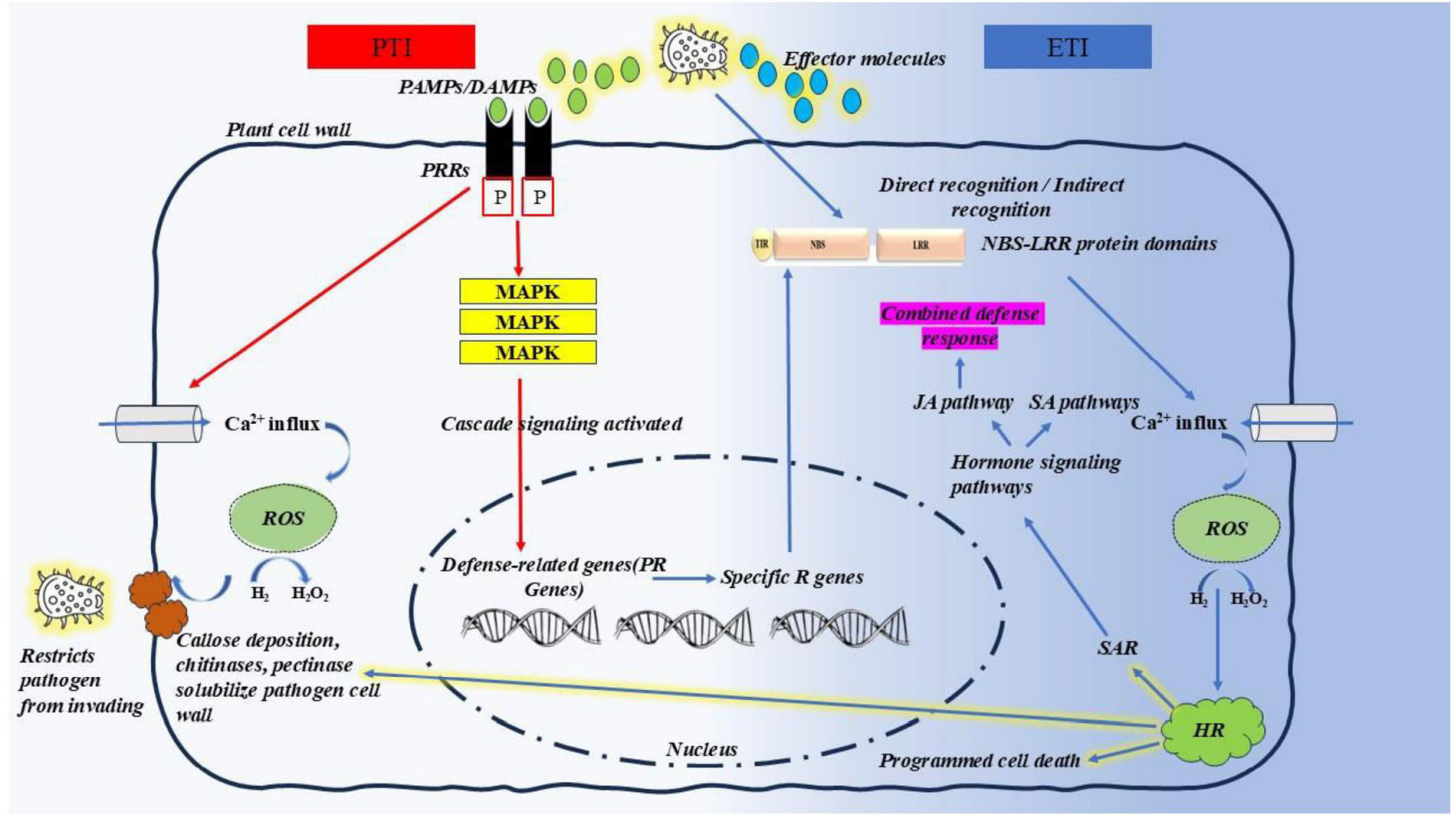
Schematic representation of plant immunity: pattern-triggered immunity (PTI) and effector-triggered immunity (ETI) (Jones et al., 2024; Yu et al., 2024; Nguyen et al., 2021; Doughari, 2015). The first line of defence, PTI (marked by red arrows), is triggered when pattern recognition receptors (PRRs) detect pathogen-associated molecular patterns (PAMPs) or damage-associated molecular patterns (DAMPs). This activates a cascade of signalling events, including mitogen-activated protein kinase (MAPK) activation, Ca^2+^ influx, and the production of reactive oxygen species (ROS). To suppress PTI, pathogens release effectors. When these effectors are recognized by nucleotide-binding (NB) and leucine-rich-repeat (LRR)-containing receptors (NLRs), the second layer of immunity, ETI (marked by blue arrows), is activated. This recognition induces conformational changes in NLRs, initiating intracellular signalling that leads to the hypersensitive response (HR) or systemic acquired resistance (SAR). SAR further activates key hormonal pathways, such as salicylic acid (SA) and jasmonic acid (JA) signalling. Research suggests that PTI and ETI are interconnected, working together to amplify immune responses (Nguyen et al., 2021).

Although PTI is primarily associated with biotrophic pathogens, several necrotrophs also produce effectors to manipulate host defences (Boller and Felix, 2009). These effectors, depending on their domains, are recognised by specific plant receptors (R proteins) encoded by R genes (Boller and Felix, 2009; Du et al., 2015; Jones and Dangl, 2006; Sarris et al., 2015). While the precise resistance mechanisms of many cloned R genes remain unclear, the most abundant R gene class encodes proteins with nucleotide-binding site (NBS) and leucine-rich repeat (LRR) domains, which play crucial roles in pathogen recognition and signalling (Dangl and Jones, 2001). NBS domains contain conserved motifs, including the P-loop, kinase-2, and Gly-Leu-Pro-Leu motifs, essential for signalling (Tan and Wu, 2012). LRRs facilitate highly adaptable protein-protein interactions, enabling diverse binding specificities (Ellis et al., 2000; Jones and Dangl, 2006). Subsequently, numerous R genes for qualitative resistance were cloned across plant species, revealing diverse amino acid motif organisations and membrane-spanning domains, which can be classified into nine distinct classes (Kourelis and Van Der Hoorn, 2018) (**Table 5**). As a second line of defence, effectors trigger downstream genes, resulting in a race-specific hypersensitive response to contain the pathogen, commonly referred to as effector-triggered immunity (ETI), qualitative resistance, or vertical resistance (Boller and Felix, 2009; Giraldo and Valent, 2013). This form of resistance is considered monogenic and gave rise to the gene-for-gene hypothesis (Flor, 1971). In ETI, cell-surface receptor-like proteins (RLPs; known as RLKs when containing a kinase domain) or intracellular NLRs detect pathogen-secreted effectors. Receptors with nucleotide-binding domains and leucine-rich repeats (NLRs) detect effectors secreted by pathogens to facilitate infection. PRRs, WAKs, and NLRs initiate signalling cascades that lead to strong resistance reactions (Kushalappa et al., 2016). Mitogen-activated protein kinases (MAPKs), G-proteins, ubiquitin, calcium, hormones, transcription factors (TFs), and epigenetic modifications regulate the expression of pathogenesis-related (PR) genes. This regulation results in various responses that prevent further infection: hypersensitive response (HR), production of reactive oxygen species (ROS), cell wall modification, stomatal closure, and the production of various anti-pest proteins and compounds (e.g., chitinases, protease inhibitors, defensins, and phytoalexins) (Andersen et al., 2018; Dangl and Jones, 2001; Niks et al., 2015; Dodds and Rathjen, 2010). Generally, PTI and ETI give rise to similar responses, although ETI is qualitatively stronger and faster and often involves a form of localized cell death called the hypersensitive response (HR). PTI is generally effective against non-adapted pathogens in a phenomenon called non-host resistance, whereas ETI is active against adapted pathogens. However, these relationships are not exclusive and depend on the elicitor molecules present in each infection (Dodds and Rathjen, 2010). The Schematic diagram of the mechanisms involved in plant immunity is represented in **Figure 4**.

**Table 5:** R genes classification mechanisms.

Mechanism	Description	R Genes (Plant Species)
1: RLP/RLK, indirect	Recognition by a modified effector of a host component, perceived by a cell surface RLK/RLP receptor.	*Cf-2* (tomato) (Kourelis and Van Der Hoorn, 2018)
2: RLP/RLK, direct	Recognition triggered by direct interactions of pathogen-derived effectors and a cell surface RLK/RLP receptors	*OsFLS2* (rice), *EFR, FLS2, LORE, LYK3, LYK4, LYK5, LYM1/LYM3, LYM2, RBGP1, RLP23* (Arabidopsis), *VvFLS2* (grapevine), *CEBiP, FLS3, LeEIX2, SlFLS2* (tomato) (Kourelis and Van Der Hoorn, 2018)
RLP/RLK, unknown mechanism		*XA21* (rice), *StoVe1* (eggplant), *Ve1* (tomato), *LepR3, RLM2* (oilseed rape), *StuVe1, ELR* (potato), *Cf-4, Cf-5, Cf-9* (tomato) (Kourelis and Van Der Hoorn, 2018)
RLK/RLP	Presence of oligogalacturonan-binding domains in the extracellular domains involved in signalling of loss of cell wall integrity	*CsLRK10L2* (cucumber-Berg et al, 2020) *OsLRR-RLP2* (Japonica Rice- Kim et al, 2024)
3: NLR, indirect	Recognition triggered either by effector binding to a host component or by effector-mediated modification of a host component, perceived by an NLR	*Rpi-blb3, Rx1, Rx2* (potato), *GmRIN4* (tobacco), *RPM1, RPS2, RPS5*(Arabidopsis), *Rpg1-b, Rpg1r* (soybean), *N*(tobacco), *Prf, Pto* (tomato) (Kourelis and Van Der Hoorn, 2018)
4: NLR, direct	Recognition is achieved by direct interaction of a pathogen derived component and an NLR receptor	*Pi-ta* (rice), *RPP1-{EstA/Nda/ZdrA}* (Arabidopsis*), L5/L6/L7, M* (flax), *Sw-5b* (tomato) (Kourelis and Van Der Hoorn, 2018) *RXL/Pm5e* (Wheat powdery mildew- Guo et al, 2025)
NLR	Positively regulates Salicylic acid and Jasmonic Acid Signalling pathways to resist TSWV.	Sl5R-1 (Tomato) *Pb2* (Rice panicle blast resistance- Ma et al, 2022)
NLR, unknown mechanism		*Rxo1* (maize), *Pi9, Pib, Piz-t*(rice), *RBA1, RCY1, RPP13-Nd-1, RPP13-UKID37, TAO1* (Arabidopsis), *Mla1, Mla10, Mla13* (barley), *Bs2* (black pepper), *P, P2* (flax),*3gG2* (soybean), *N* (tobacco), *Bs4, I2, Tm-2, Tm2^2^* (tomato) (Kourelis and Van Der Hoorn, 2018)
5: NLR-ID	Recognition is triggered either by effector binding to a domain or by effector-mediated modification of a domain that is integrated in a host NLR.	*Pii-2, Pik-{1/h/p1/s}, RGA5-A, Xa1* (rice), *RRS1B, RRS1-R, RRS1-S* (Arabidopsis), R1 (potato) (Kourelis and Van Der Hoorn, 2018)
6: Executor	Recognition triggered by transcriptional activation of the executor gene by a pathogen TAL effector.	*Xa7, Xa10, Xa23* (rice), *Bs3, Bs3-E, Bs4C-R* (pepper- Kim and Hwang, 2015), *Rph3* (Barley leaf rust-Dinh et al, 2022)
7: Other, Passive	Loss of susceptibility by mutation in a host component, leading to the inability to manipulate the host.	*xa13, xa25, xa5* (rice), *Tsn1, Snn1* (wheat), *rwm1, lov1* (Arabidopsis), *Rym-4, Rym-5* (barley), *retr01*(cabbage), *bc-3* (French bean), *Mo-1* (lettuce), *sbm1* (pea), *Eva1* (potato), *LGS1, Pc* (sorghum), *Asc-1, Ty-5, Pot-1*(tomato) (Kourelis and Van Der Hoorn, 2018)
8: Other, active	Loss of susceptibility by directly disarming the pathogen by actively interrupting a key pathogenicity process.	*STV11-R* (rice), *Tm-1, Ty-1, Ty-3* (tomato), *RTM1, RTM2, RTM*(Arabidopsis), *Hv, Hm1* (barley), *Hm1, Hm2, ZmTrxh* (maize) (Kourelis and Van Der Hoorn, 2018)
Other	Serine/threonine dual specificity protein kinase. Candidate for *Rlm1* black leg resistance gene	*BnaA07g27460D* (Brassica napus- Fu et al, 2019)
9: Reprogramming	Loss of susceptibility by a deregulated host.	*Lr34, Lr67, YrL693, Yr36* (wheat), *GH3-2, GH3-8, pi21* (rice), *mlo* (barley), (Kourelis and Van Der Hoorn, 2018)

Modified from Kourelis and Van Der Hoorn (2018).

In 2016, Kushalappa et al. proposed a unifying concept of plant resistance, describing it as a continuum of reduced susceptibility, ranging from complete susceptibility to hypersensitive response - often referred to as “shades of grey.” According to this concept, the hypersensitive response (HR) or cell death is considered qualitative resistance, while the remaining spectrum of reduced susceptibility falls under quantitative resistance. Quantitative resistance can be measured through monocyclic processes under greenhouse conditions, including infection efficiency, latent period, lesion expansion, and sporulation, or polycyclic processes in field conditions, such as the apparent infection rate and the area under the disease progress curve (Kushalappa and Gunnaiah, 2013). Plant resistance to pathogen stress is controlled by a hierarchy of genes, designated as R genes with subscripts based on their functions. These genes regulate the production of resistance-related metabolites (RRMs) and resistance-related proteins (RRPs), which either suppress pathogens through antimicrobial activity or reinforce cell walls to contain infections. Furthermore, the distinction between qualitative and quantitative resistance, as well as between PTI and ETI, is not always absolute; rather, they exist along a continuum with overlapping mechanisms (Poland et al., 2009).

## Cloning and characterisation of resistance-related genes/genomic regions

**Figure 5:**
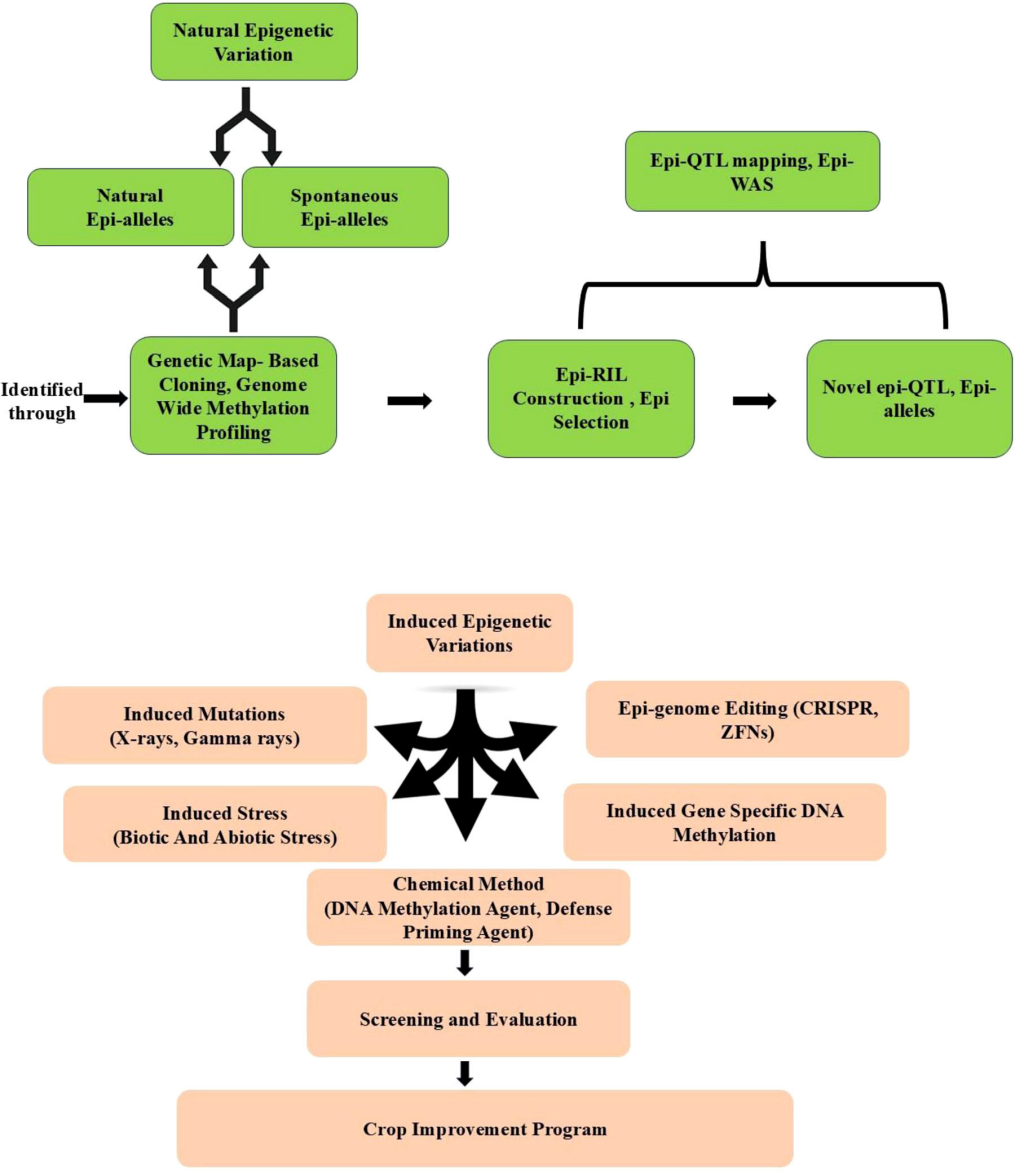
Epi-breeding design for crop disease resistance improvement. Epigenetic variations are either derived from natural populations, or induced by stresses, chemical treatments, mutations in epigenetic machinery, induced gene-specific DNA methylation, and epigenome editing (Zhi and Chang, 2021).

In plants, resistance (*R*) genes play a crucial role in conferring specific resistance and have proven valuable in many breeding programs. The first cloned *R* gene, *Hm1*, from maize (*Zea mays* L.), encodes an enzyme that detoxifies the *Helminthosporium carbonum* (HC) toxin produced by the fungal pathogen *Cochliobolus carbonum* (Johal and Briggs, 1992). *Hm1* encodes a reduced nicotinamide adenine dinucleotide phosphate (NADPH)-dependent HC-toxin reductase, which inactivates HC-toxin, a pathogenicity factor secreted by *C. carbonum*, allowing the fungus to infect specific maize genotypes. However, the genetic interaction between maize and *C. carbonum* deviates from the classic gene-for-gene model, as toxin-deficient *C. carbonum* strains lose their virulence even in maize cultivars lacking *Hm1*. The first *R* gene cloned under a classic gene-for-gene interaction was the tomato *Pto* gene, which confers resistance to *Pseudomonas syringae* pv. *tomato* (*Pst*) strains carrying the avirulence gene *avrPto* (Martin et al., 1993). Subsequent *R*gene cloning efforts led to the identification of *Cf-9* from tomato (*Solanum lycopersicum*) (Jones et al., 1994), *N* from tobacco (*Nicotiana tabacum*) (Witham et al., 1994), and *RPS2* from *Arabidopsis thaliana* (Bent et al., 1994; Mindrinos et al., 1994). To date, over 450 *R* genes have been identified, playing crucial roles in plant immunity, with most encoding nucleotide-binding site (NBS)–leucine-rich repeat (LRR) proteins (Chen et al., 2024) (**Figure 5**). In crop plants, the identification of quantitative trait loci (QTLs) is often facilitated by linkage analysis and genome-wide association studies (GWAS) to locate genomic regions associated with resistance phenotypes. Over time, improved resolution has refined QTL mapping, narrowing down causal loci. The choice of QTL mapping strategy depends on the available mapping population and genetic resources. Advances in genome sequencing have accelerated the identification of large-effect quantitative resistance loci (QRLs), offering significant potential for marker-assisted selection (Thakur et al., 2023). Some major QTLs/*R* genes are listed in **Table 6**.

**Table 6:** QTLs/genes identified in major and minor crops.

Crop	Disease	QTLs/Major genes identified	Chromosomal location	References
Rice	Bacterial Panicle Blight	*qBPB-1-1, qBPB-1-2*	1	Pinson et al., 2010
	Blast	*Pi-sh, Pi-9*	1,6	Imbe and Matsumoto, 1985; Takahashi et al., 2010; Amante-Bordeos et al., 1992, Qu et al., 2006
	Sheath Blight	*qSBR11-1, qSBR1-2*	1	Fu et al., 2011
Wheat	Leaf rust	*Lr2K38/QLr.ags-1AL*, Lr*37, Lr 34*	1AL, 2AS, 7DS	Sapkota et al., 2020; Helguera et al., 2003; Lagudah et al., 2006
	Stripe rust	*Yr18*, *Yr5*	7DS, 2B	Lagudah et al., 2006; Wan et al., 2004
Maize	Northern leaf blight	*Ht1*, *Ht2*	2L, 8	Simcox and Bennetzen, 1993; Yin et al., 2003
Soybean	Soybean cyst nematode	*Rhg1, Rhg4*		Ruben et al., 2006
	Phytophthora root rot	*Rps3a, Rps3b, Rps3c, rps8*	13L	Gunadi, 2012
Potato	Late blight	*Rpi-vnt1*	9	Pel et al., 2009
Tomato	Bacterial wilt	*Bwr-6, Bwr-12*	6, 12	Wang et al., 2013
	Late blight	*Ph-3, Ph-2, Ph-1*	9,10,7	Shekasteband et al., 2015; Scott and Gardner, 2007; Foolad et al., 2004
Pea	Root rot	Ae-Ps7.6	Chromosome 7	Wu et al., 2021
Chick Pea	Fusarium Wilt	*Foc-4*	Chromosome 2	Bhutia et al., 2024
Finger Millet	Blast	*ELECO.r07.8BG0647230* *ELECO.r07.8G0647240*	8B	Pendergast et al., 2022

Currently, more than 95 resistance genes have been identified in rice (*Oryza sativa*) (Koide et al., 2009). Among these, *Pi-1(t)*, *Pi2*, *Pi9*, *Pi20(t)*, *Pi27(t)*, *Pi39(t)*, *Pi40(t)*, and *Pikh* confer broad-spectrum resistance (BSR) (Liu et al., 2002; Zhu et al., 2004; Jeung et al., 2007; Liu et al., 2007; Li et al., 2008; Yang et al., 2008). Additionally, hundreds of QTLs associated with quantitative resistance to blast disease have been identified, though most remain uncharacterized at the molecular level (Ballini et al., 2008; Fukuoka et al., 2009; Ashkani et al., 2015). The complexity of QTL effects, along with genetic and environmental interactions, has limited the fine-mapping of resistance loci (Fukuoka et al., 2015). Genetic studies on the *rice–Magnaporthe oryzae* interaction have identified nearly 100 race-specific resistance genes and over 350 QTLs across the rice genome, except chromosome 3 (Ashkani et al., 2015). A newly emerging oat disease caused by *Pyrenophora avenicola*, responsible for leaf spot, is becoming a major concern. Identifying QTLs associated with resistance could provide a strategic framework for genetic improvement and marker-assisted selection. A study represents a milestone in oat genetics by reporting the first putative QTLs linked to *Pyrenophora* leaf spot resistance in oats (*Avena sativa*).

A meta-analysis of 16 mapping experiments for rice diseases identified 94 QRLs, covering over half of the rice genome (Wisser et al., 2005). Similarly, Devanna et al. (2024) identified 199 putative rice blast resistance genes within 53 meta-QTL (MQTL) regions, including 48 characterised resistance gene analogues (RGAs) and related proteins, such as NBS-LRR receptors, LRR receptor-like kinases, NB-ARC domain proteins, and pathogenesis-related transcription factors (TF/ERF domain). These also include proteins involved in elicitor-induced defence and defence signalling. In maize, a synthesis of 50 studies identified 437 QRLs, covering 89% of the genome (Wisser et al., 2006). While the resistance mechanisms of most QRLs remain unknown, some have been fine-mapped and cloned. Occasionally, multiple linked genes, including clusters of defence-related genes involved in secretory processes and cell wall reinforcement, are found within a single QTL (Wisser et al., 2005).

## Wild germplasm: a treasure trove for crop improvement

A key cornerstone of any crop resilience breeding program is the availability of diverse germplasm, contributed by cultivars grown in different geographical regions, unimproved landraces, and wild crop relatives. One of the most notable examples is the 1BL/1RS translocation in “Veery” wheat, which introduced resistance to three rust diseases and powdery mildew through the incorporation of a rye (*Secale cereale*) chromosome arm. This segment carries the genes *Lr26*, *Sr31*, *Yr9*, and *Pm8*, conferring race-specific resistance to leaf rust, stem rust, stripe rust, and powdery mildew, respectively (Ren et al., 2018). Similarly, the transfer of the bacterial leaf blight (*BLB*) resistance gene *Xa21* from *Oryza longistaminata* marked a breakthrough in disease resistance research in rice. This gene provided broad-spectrum resistance against multiple races of *Xanthomonas oryzae* pv. *oryzae* in both India and the Philippines (Savary et al., 2019). In maize, the southern corn leaf blight (*SCLB*), caused by *Bipolaris maydis* race T (formerly *Helminthosporium maydis* T), reached epidemic proportions in the United States and southern Canada, leading to an estimated 15% crop loss, valued at $1 billion at that time (Ullstrup, 1972). The crisis was mitigated by introgressing blight-resistant alleles from a wild relative of Mexican maize, *Tripsacum dactyloides*, into commercial maize lines (Maxted and Kell, 2009). Thus, exploring and utilising wild genetic resources remains a key pre-breeding strategy, as these wild relatives serve as potential sources of disease-resistant genes. In maize, one of the most commonly utilised wild germplasm sources is *Zea mays* subsp. *parviglumis* (teosinte), which is recognised as a primary genetic resource due to its wide allelic diversity. This diversity has contributed to resistance against several major maize diseases, including gray leaf spot (*Cercospora zeae-maydis*) and northern corn leaf blight (*Exserohilum turcicum*) (Wani et al., 2022). Lennon et al. (2016) also identified specific alleles in teosinte that confer effective resistance against these pathogens, endorsing the potential for transferring beneficial traits from wild relatives to cultivated maize varieties. In this review, we summarise the utilisation of wild resources for disease resistance in crops (**Table 7**).

**Table 8:** List of successful genome edited varieties.

Crop	Gene	Genome Editing method	Disease Resistance	References
Wheat	*MLO*	CRISPR-cas9, TALEN	Powdery Mildew	Wang et al., 2014
Rice	*OsSWEET14*	TALEN	Bacterial blight	Li et al., 2012
Cucumber	*eIF4E*	CRISPR-cas9	Resistance to potyviruses	Chandrasekaran et al., 2016
Tomato	*SlPMR4*	CRISPR-cas9	Powdery mildew	Nekrasov et al., 2017
Banana	*MusaPDK*	CRISPR-cas9	Fusarium wilt	Tripathi et al., 2019
Potato	*StGBSSI*	CRISPR-cas9	Late blight	Ghislain et al., 2019
Grape Vine	*VvWRKY52*	CRISPR-cas9	Downy mildew	Wang et al., 2020
Soybean	*GmSWEET10a*	CRISPR-cas9	Bacterial pathogens	Cai et al., 2020
Maize	*ZmCKX1*	CRISPR-cas9	Fusarium ear rot	Wang et al., 2020
Rice	*OsERF922*	CRISPR-cas9	Rice blast	Wang et al., 2016
Tomato	*SlJAZ2*	CRISPR-cas9	Tomato yellow leaf curl virus	Pramanik et al., 2021
Barley	*HvMORC1*	CRISPR-cas9	Powdery mildew	Galli et al., 2022
Cotton	*GhMLO*	CRISPR-cas9	Verticillium wilt	Umer et al., 2023

## Breeding strategies for developing disease resistance

### Conventional breeding methods

Breeding for durable and broad-spectrum disease resistance is fundamental to commercial breeding programs for biotic stress management. A crop’s genetic structure significantly influences its disease susceptibility. Self-pollinated crops (e.g., wheat, barley, oats, peas) are highly uniform due to homozygosity, making them vulnerable to diseases. Similarly, asexually propagated clonal cultivars (e.g., potato, strawberry, banana, fruit trees) and single-cross hybrids exhibit uniformity, increasing their disease susceptibility. Vegetative propagation units (e.g., tubers, bulbs, cuttings) can also harbour pathogens across growing seasons. In contrast, cross-pollinated species and three-way/double-cross hybrids maintain higher genetic diversity, enhancing their buffering capacity against diseases. Despite this, most commercial crops remain genetically uniform, making them prone to disease outbreaks. Breeding programs are tailored to specific crops, pathogens, and environmental conditions, yet the primary goal remains the accumulation of favourable resistance genes in elite cultivars. Landraces, wild relatives, and induced mutations serve as valuable resistance sources for commercial breeding. Conventional breeding methods, such as pedigree selection, backcross breeding, mutation breeding, and recurrent selection, have long been used to develop disease-resistant cultivars. Among these, backcross breeding has been particularly successful in transferring resistance traits (Hussain, 2015). For instance, bacterial blight resistance in rice (*Oryza sativa*) was introduced by transferring *Xa21* from *Oryza longistaminata* through backcrossing, resulting in the IRBB lines (Huang et al., 1997). In wheat (*Triticum aestivum*), genes such as *Lr24* and *Lr26* were introgressed to enhance leaf rust resistance (Singh et al., 2011). In tomato (*Solanum lycopersicum*), resistance to tomato mosaic virus (ToMV) was introgressed from *Solanum peruvianum*, marking a milestone in tomato breeding (Pilowsky and Cohen, 1974).

While backcross and pedigree selection are widely used in self-pollinated crops, recurrent selection has been effective in cross-pollinated species by accumulating favourable alleles over successive cycles (Miedaner, 2016). In maize, recurrent selection has enhanced resistance to northern leaf blight (*Exserohilum turcicum*), yielding notable genetic gains (Scheiner et al., 2020). In soybean (*Glycine max*), it has improved resistance to soybean cyst nematode (*Heterodera glycines*), while in perennial ryegrass (*Lolium perenne*), recurrent selection has developed crown rust-resistant (*Puccinia coronata*) populations (Concibido et al., 2004; Wilkins and Humphreys, 2003). However, a single resistance gene may not provide durable protection due to pathogen evolution. Gene pyramiding, a concept dating back to traditional plant breeding (Kaushik et al., 2016), combines multiple resistance genes to enhance durability and broaden resistance spectra. This approach has successfully introduced major-effect resistance genes into elite cultivars, making it harder for pathogens to overcome resistance. In conventional breeding, gene pyramiding is achieved by selecting resistant sources and incorporating them into high-yielding backgrounds through repeated backcrossing.

Despite its effectiveness, conventional breeding is slow, labour-intensive, and costly. Developing new varieties via backcross breeding is time-consuming and often results in rapid resistance breakdown due to pathogen evolution. Additionally, introgressing QTLs from non-elite germplasm while minimising linkage drag remains a challenge (Riseh and Vazvani, 2024). To accelerate breeding, molecular marker technologies such as marker-assisted selection (MAS) has revolutionised resistance breeding, allowing for precise gene identification and introgression (Torres, 2009; Hafeez et al., 2023; Jabran et al., 2023; Nelson et al., 2018).

### Advanced breeding methods

Advancements in plant genomics and biotechnology have enabled breeders to develop cultivars with multi-gene resistance to biotic stresses. Introgressing QTLs from non-elite germplasm while minimising linkage drag remains a challenge in conventional breeding (Riseh and Vazvani, 2024). To accelerate breeding, molecular marker technologies such as marker-assisted selection (MAS) has revolutionized resistance breeding, allowing for precise gene identification and introgression (Torres, 2009; Hafeez et al., 2023; Jabran et al., 2023).

Gene pyramiding via MAS has been widely used to assemble resistance genes from different sources. Fine mapping of *R* genes and QTLs using association mapping and genome-wide association studies (GWAS) has improved marker-assisted breeding (MAB) (Mapari and Mehandi, 2024). A significant success story is the development of bacterial blight-resistant rice cultivars, where *Xa21* was introgressed from wild rice using MAS (Khush et al., 1990). In wheat, MAS facilitated the pyramiding of leaf rust resistance genes *Lr13, Lr34*, and *Lr37* (Kloppers and Pretorius, 1997), as well as powdery mildew resistance genes *Pm2* and *Pm4a* (Liu et al., 2000; Wang et al., 2001).

However, until recently, the lack of genetic markers made it difficult to introgress resistance genes due to linkage drag and unexplored genetic diversity (Sánchez-Martín and Keller, 2019). This was overcome by next-generation sequencing (NGS) and high-throughput genotyping, which have reduced costs and improved association mapping (Sánchez-Martín and Keller, 2019). GWAS now allows the rapid identification of complex resistance traits, significantly reducing fine-mapping time compared to traditional QTL mapping (Dubey and Mohanan, 2025). Recently, Saxena et al. (2025) identified 12 QTLs for blast resistance in rice, revealing candidate genes encoding NBS-LRR receptors, protein kinases, and pathogenesis-related proteins.

Additionally, transcriptomic and proteomic analyses and metabolomics have provided deeper insights into dynamic resistance regulation, aiding in the identification of regulatory networks contributing to qualitative and quantitative resistance (Choi et al., 2022). In order to understand the molecular components underlying plant pathogen, gene expression profiling was first applied to *Arabidopsis thaliana* (Schenk et al., 2000). In this study, changes in the expression patterns of more than 2300 selected genes were examined simultaneously by cDNA microarray analysis in Arabidopsis after inoculation with the fungal pathogen *Alternaria brassicicola*. Results showed the existence of a substantial network of regulatory interactions and coordination events occurring during plant defence among the different defence signalling pathways including interactions between the salicylate and jasmonate pathways. Many breeding programs rely on creating variation in elite lines, yet the erosion of genetic diversity remains a concern. Non-elite germplasm contains valuable resistance traits, but undesirable linkage drag poses challenges in breeding pipelines. Transgenic and genome editing technologies now offer new possibilities for disease resistance breeding by transferring *R* genes, modifying quantitative disease resistance (*QDR*), and altering susceptibility (*S*) genes. Successful transgene-based resistance has been demonstrated across multiple crop species (Dangl et al., 2013). For example, transgenic maize expressing rnc70, an *Escherichia coli* gene, exhibited reduced infection by rice black-streaked dwarf virus (RBSDV) in field trials (Cao et al., 2013). Similarly, in cassava, transgenic lines conferring resistance to two cassava viruses were combined with natural resistance to cassava mosaic disease (Vanderschuren et al., 2012). However, transgenic disease-resistant varieties remain rare due to intellectual property issues, regulatory constraints, technical challenges, and public concerns (Collinge and Sarrocco, 2022; Lucas, 2011; Scott et al., 2016; Fuchs and Gonsalves, 2007).

Another emerging strategy is genomic selection (GS), which enables breeders to select for small-effect resistance alleles using high-throughput genotyping (Poland and Rutkoski, 2016). GS is particularly useful when phenotypic data are costly or difficult to obtain (Heffner et al., 2010). It has been successfully applied to improve disease resistance in wheat, maize, and cassava (Poland and Rutkoski, 2016; Jangra et al., 2021). The use of high-throughput phenotyping (HTP) has further revolutionised resistance breeding by enabling real-time screening of disease resistance traits across thousands of genotypes (Sankaran et al., 2015; Freitas Moreira et al., 2021). Remote sensing, automated image analysis, and machine learning algorithms have enhanced precision in tracking plant-pathogen interactions (Rebetzke et al., 2019; Li et al., 2014; Yu et al., 2016).

In plant immune regulation, epigenetic modifications, such as histone methylation, acetylation, ubiquitination and DNA methylation, and demethylation, play crucial role in R gene-mediated immunity (Xie and Duan, 2023). With the contribution of DNA (de)methylation mutants and the advancement of DNA methylation profiling techniques—including methylation-sensitive amplified fragment length polymorphism (MSAP) analysis, whole-genome bisulfite sequencing (WGBS), methylated DNA immunoprecipitation sequencing (MeDIP-seq), and methyl-CpG binding domain protein capture sequencing (MBDCap-seq)—the dynamics and biological functions of DNA (de)methylation in plant-pathogen interactions have been extensively studied in both model and crop plants (Clark et al., 1994; Guevara et al., 2017; Li et al., 2018; Feng and Lou, 2019; Tirnaz and Jacqueline, 2019; Hsu et al., 2020). Histone acetylation is pivotal in plant immunity; in rice, the HD2 subfamily histone deacetylase HDT701 functions as a negative regulator by modulating histone H4 acetylation of DR genes against bacterial blight (Ding et al., 2012). In wheat, the histone deacetylase TaHDA6 interacts with TaHOS15 and is recruited to the promoter of R genes, including TaPR1, TaPR2, TaPR5, and TaWRKY45, fine-tuning resistance to powdery mildew (Zhi et al., 2020). Histone ubiquitination also influences plant immunity by modulating JA, SA, and ET hormone signalling pathways (Gao et al., 2022). Hence, Epigenetic factors serve as key regulators in the transcriptional reprogramming of plant immune responses, suggesting that epigenetics-based strategies can be broadly employed to enhance plant disease resistance. Increasing evidences proving that DNA methylation and histone modifications are involved in transgenerational systemic acquired resistance (SAR) (Slaughter et al., 2012; He and Li, 2018; Stassen et al., 2018; Sharrock and Sun, 2020). Therefore, epi-breeding referring to the breeding for epigenetic changes provides new avenues for crop resistance improvement. Both natural and artificially induced epigenetic variations can influence plant disease resistance and have great potential in epi-breeding for crop resistance improvement. Furthermore, epigenetic variants could be experimentally obtained by chemical treatments, mutations in epigenetic machinery, induced gene-specific DNA methylation, and epigenome editing (Varotto et al., 2020) (**Figure 5**).

## Genome editing: a cutting-edge approach to accelerate disease resistance

Traditional breeding for disease resistance is inherently time-consuming, requiring repeated backcrossing to introduce desirable traits while getting rid of linkage drag. Genome editing has revolutionized this process by enabling precise and direct modification of target genes within elite crop varieties, significantly accelerating trait improvement (Pixley et al., 2022). Target-specific gene silencing has gained immense popularity among the scientific community over the years. Among various gene-silencing techniques, RNAi has proven highly effective in targeting and degrading specific mRNAs. Through the RNAi pathway, long double-stranded RNAs (dsRNA) are processed into small interfering RNAs (siRNA), which bind and cleave targeted viral messenger RNAs (mRNA) in the cytosol, thereby conferring effective plant protection (Mezzetti et al., 2020). This versatile tool is being widely utilized to disrupt pest and pathogen genes via host-induced gene silencing (HIGS) (Nowara et al., 2010) and spray-induced gene silencing (SIGS) (Koch et al., 2016). In barley, *Fusarium graminearum* infection was controlled by spraying a 791-nucleotide-long dsRNA (CYP3-dsRNA) (Koch et al., 2016).

Beyond RNAi, genome editing technologies—particularly CRISPR/Cas systems—have ushered in a new era of precision breeding for disease resistance. Unlike RNAi, which acts post-transcriptionally by degrading viral RNA, CRISPR/Cas9 enables the direct and permanent modification of plant genomes, allowing for the targeted knockout of susceptibility genes or the precise insertion of resistance-conferring alleles. This approach not only provides durable and heritable resistance but also circumvents the limitations of conventional breeding and transgenic approaches (Silva and Fontes, 2022; Khan et al., 2022). Genome editing relies on site-directed nucleases (SDNs), including zinc-finger nucleases (ZFNs), transcription activator-like effector nucleases (TALENs), and CRISPR–Cas9, to introduce precise genetic changes (Lusser et al., 2011) (**Table 8**). The growing availability of genome sequencing data has facilitated the identification of gene targets for editing, enabling researchers to delete, modify, or insert genes to improve disease resistance (Wang et al., 2023). For example, TALEN-mediated gene disruption of a sucrose efflux transporter enhanced bacterial blight resistance in rice, while CRISPR–Cas9-induced mutations in an ethylene response factor conferred blast resistance (Vázquez-García et al., 2021). Among all, the CRISPR-Cas system has emerged as one of the most advanced and precise methods for genetic manipulation due to its simplicity, high efficiency, and versatility (Gao, 2021; Greenwood et al., 2023; Kumar et al., 2020). Various CRISPR-Cas tools have been developed for a range of genetic modifications, including targeted gene knockout, gene insertion and replacement, base editing, epigenome editing, and CRISPR mediated transcriptional regulation. Additionally, a novel genome editing tool based on a transposon-associated RNA-guided endonuclease known as TnpB, considered the ancestor of Cas12, has been engineered for genome editing (Karvelis et al., 2021).

**Table 8:** List of successful genome edited varieties.

Crop	Gene	Genome Editing method	Disease Resistance	References
Wheat	*MLO*	CRISPR-cas9, TALEN	Powdery Mildew	Wang et al., 2014
Rice	*OsSWEET14*	TALEN	Bacterial blight	Li et al., 2012
Cucumber	*eIF4E*	CRISPR-cas9	Resistance to potyviruses	Chandrasekaran et al., 2016
Tomato	*SlPMR4*	CRISPR-cas9	Powdery mildew	Nekrasov et al., 2017
Banana	*MusaPDK*	CRISPR-cas9	Fusarium wilt	Tripathi et al., 2019
Potato	*StGBSSI*	CRISPR-cas9	Late blight	Ghislain et al., 2019
Grape Vine	*VvWRKY52*	CRISPR-cas9	Downy mildew	Wang et al., 2020
Soybean	*GmSWEET10a*	CRISPR-cas9	Bacterial pathogens	Cai et al., 2020
Maize	*ZmCKX1*	CRISPR-cas9	Fusarium ear rot	Wang et al., 2020
Rice	*OsERF922*	CRISPR-cas9	Rice blast	Wang et al., 2016
Tomato	*SlJAZ2*	CRISPR-cas9	Tomato yellow leaf curl virus	Pramanik et al., 2021
Barley	*HvMORC1*	CRISPR-cas9	Powdery mildew	Guan et al., 2022
Cotton	*GhMLO*	CRISPR-cas9	Verticillium wilt	

**Figure 6:**
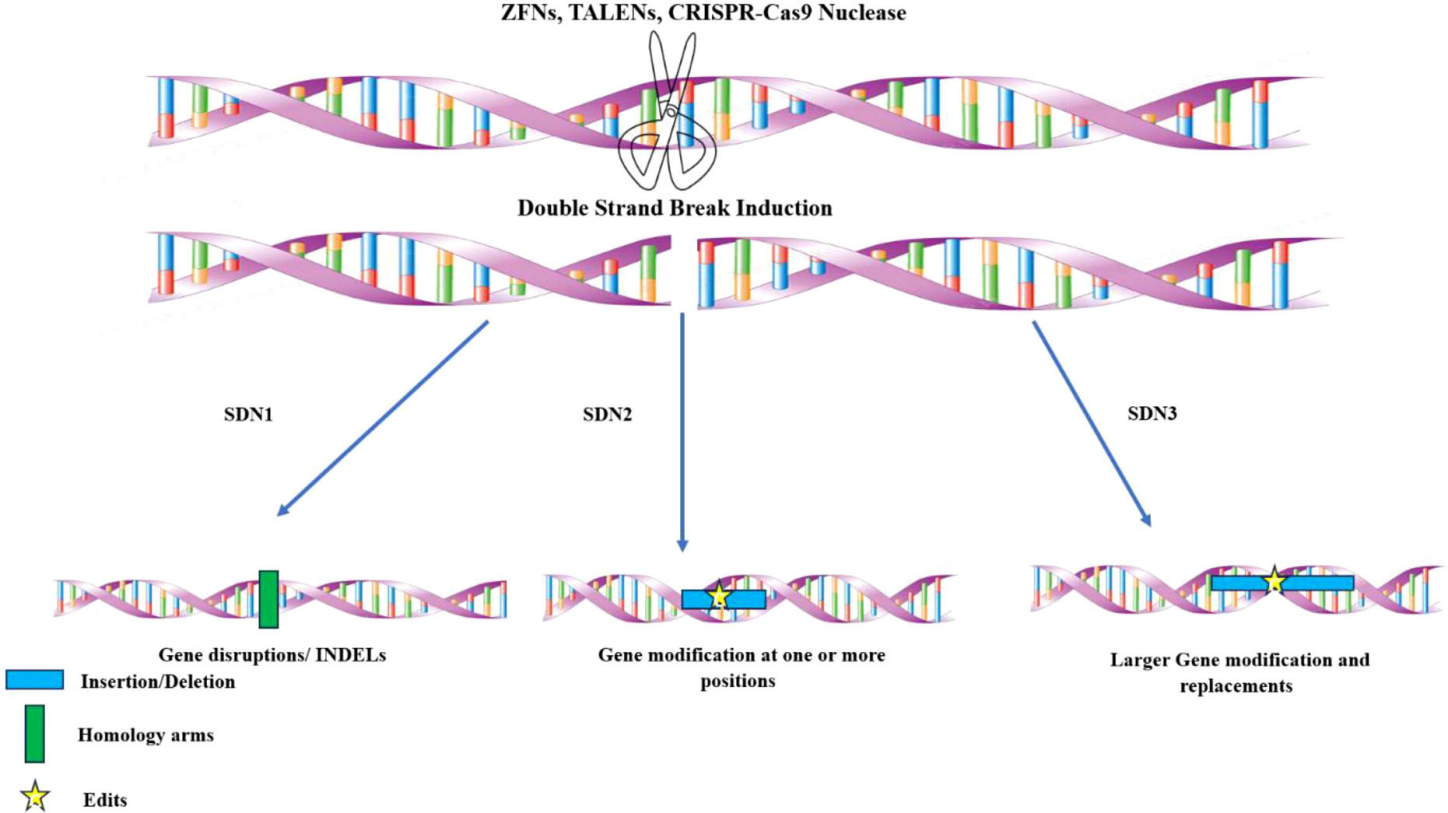
Schematic diagram of SDN1, SDN2, and SDN3. Nucleases such as ZFNs, TALENs, and CRISPR/Cas9 bind with target DNA to cause DSBs that are repaired by two different mechanisms. SDN1 does not need a template and results in gene disruptions through indels (small insertions or deletions of bases). SDN2 utilizes a homologous template and results in gene correction or modification at one or more positions. SDN3 requires a full gene as a template, and leads to gene replacement or foreign DNA insertion.

Three main recent genome editing strategies have been recognised:

- SDN-1 – Induces small, random mutations without a template, leading to gene knockouts.
- SDN-2 uses a short DNA template to introduce precise sequence changes through homology-directed repair (HDR).
- SDN-3 – Inserts larger DNA segments (e.g., entire genes) via HDR.
- Among these, SDN-2/3 allele swaps offer great potential for rapid deployment of disease resistance genes in elite varieties while maintaining the native genetic background. A notable example is the allele swap in rice, where a small gene fragment transferred the elite *indica* allele of *NRT1.1B* into *japonica* rice in a single generation (Li et al., 2018) (**Figure 6**).
- Beyond gene knockouts and allele swaps, genome editing also enables precision modifications of disease resistance genes. For instance:
- Pattern recognition receptors (PRRs) can be engineered to recognize a broader range of pathogens. The Brassicaceae *Ef-Tu* receptor gene was successfully transferred to Solanaceous plants, enhancing their resistance to bacterial pathogens (Lacombe et al., 2010).
- Leucine-rich repeat (LRR) regions of *R* genes can be modified to alter pathogen recognition, as demonstrated in the mildew resistance locus *Mla* gene family in cereals (Maekawa et al., 2019).
- NLR-integrated domains (NLR-IDs), which mediate effector recognition, can be engineered or replaced to enhance immune response. For example, modifying the HMA domain of *Pik-1* altered its specificity for *Magnaporthe oryzae* effectors (De la Concepcion et al., 2018).

Genome editing holds immense potential for creating customized disease-resistant crops, but challenges remain. Not all synthetic *R* genes perform effectively in field conditions, necessitating further research into regulatory networks and gene interactions (Cesari et al., 2022).

Additionally, transgenic plants expressing Cas9 and single guide RNAs (sgRNAs) have been engineered to neutralise gemini virus sequences, conferring viral resistance (Choi et al., 2025). Transgenic approaches have significantly expedited the accumulation of desired traits in plants, particularly in enhancing disease resistance. For instance, to combat wheat stem rust, a gateway recombinase cloning strategy was employed to construct a gene cassette containing five R genes: the race-specific resistance genes *Sr22, Sr35, Sr45, Sr50* and the multi-pathogen resistance gene *Sr55*. This cassette was transformed into wheat at a single locus, enabling rapid gene stacking and conferring BSR against both stem rust and leaf rust (Luo et al., 2021). With its unparalleled precision, efficiency, and adaptability, gene editing is poised to redefine crop protection strategies, making plants more resilient to evolving biotic stresses and ensuring global food security.

## Strategies for developing broad-spectrum disease resistance

For a disease resistance breeding programme to be successful, understanding plant defence mechanisms and leveraging genomic technologies is essential for designing new resistance genotypes. Generating durable broad-spectrum resistance (BSR)—which protects against multiple pathogens-is a key objective in modern breeding. Combining multiple R genes and/or QRLs within a single genome enhances resistance durability. The durability of resistance depends on various factors, including the biology, genetics, and evolutionary adaptability of the pathogen (McDonald and Linde, 2002). Monogenic resistance based on a single *R* gene is often short-lived, as pathogens rapidly evolve to evade recognition (Parlevliet, 2002), particularly when resistant cultivars are grown in large monocultures. However, not all *R* genes are equally vulnerable to being overcome. The likelihood of an *R* gene maintaining effectiveness depends on the evolutionary constraints imposed on the pathogen. Some pathogens incur a fitness penalty when mutating to evade recognition, a phenomenon known as the cost of virulence (Huang et al., 2006). The stability of an *R* gene is linked to the functional importance of the effector it recognises. If an effector is essential for pathogen survival or virulence, mutations may be less likely to persist (Boyd et al., 2013). Identifying such conserved effectors, either by their high levels of conservation across pathogen populations or their interaction with key plant proteins, could improve the prediction of *R* gene durability (Leach et al., 2001; Pietravalle et al., 2006; Mukhtar et al., 2011). Although BSR is primarily conferred by QRLs, some qualitative resistance genes have also been associated with long-term effectiveness. For example, the wheat gene Lr*34* has provided multi-pathogen resistance for over a century (Krattinger et al., 2009). Unlike typical *R* genes, *Lr34* encodes an ATP-binding cassette (ABC) transporter, contributing to partial resistance rather than a strong immune response. *Lr34* encodes an ATP-binding cassette (ABC) transporter rather than an NLR protein and provides incomplete but durable disease resistance. Although typically classified as an *R* gene, it is functionally considered a strong QRL due to its long-lasting, broad-spectrum effectiveness (Krattinger et al., 2009; Nelson et al., 2018). Predicting *R* gene durability may be possible by understanding its cognate effector (Leach et al., 2001; Pietravalle et al., 2006). *R* genes are more likely to remain effective if they target effectors that are essential for pathogen survival or virulence (Boyd et al., 2013). Such essential effectors can be identified based on their high conservation across pathogen populations or their interaction with key plant proteins (Mukhtar et al., 2011). While applying these principles in breeding programmes is not yet standard practice, advancements in effector biology and genomic technologies are rapidly improving our ability to identify durable *R* genes with long-term effectiveness against evolving pathogen threats.

## Susceptibility genes: negative regulators of plant immunity

While *R* genes mediate pathogen recognition, susceptibility (*S*) genes facilitate infection by enabling pathogen compatibility, often through haustoria formation for nutrient acquisition. Mutating or disabling *S* genes can impair pathogen success, conferring resistance (Van Schie and Takken, 2014). Unlike dominant *R* genes, *S* gene-mediated resistance is generally recessive and can be pathogen-specific (by blocking infection mechanisms) or broad-spectrum (by triggering prolonged defence responses), though the latter may incur fitness costs (Van Schie and Takken, 2014). Despite the widespread use of *R* genes, their effectiveness is often short-lived due to pathogen evolution (Lapin and Van den Ackerveken, 2013; Wang and Valent, 2017). A promising alternative is modifying *S* genes, which removes the host factors essential for infection, limiting pathogen adaptability (Van Schie and Takken, 2014). Two well-characterised *S* genes, Mlo and *eIF4*, highlight their broad applicability across crops. Recessive *mlo* alleles in barley (*Hordeum vulgare*) provide durable powdery mildew resistance by disrupting pathogen-supporting functions. Similarly, mutations in *eIF4*, a translation initiation factor required for potyvirus infection, impair viral RNA translation, conferring resistance (Schmitt-Keichinger, 2019; Garcia-Ruiz et al., 2021). Genome editing has accelerated *S* gene-based resistance breeding. CRISPR/TALEN-mediated knockout of all three *Mlo* alleles in hexaploid wheat resulted in broad-spectrum resistance to powdery mildew. Similarly, targeted mutations in *SWEET11, SWEET13, and SWEET14* conferred resistance to bacterial blight in rice (Eom et al., 2019; Oliva et al., 2019).

While *S* gene editing offers a novel strategy for durable resistance, potential trade-offs exist, as some *S* genes also regulate plant growth and stress responses. Future strategies may focus on fine-tuning *S* gene expression rather than complete knockouts to balance resistance and agronomic performance (Li et al., 2020).

## Growth-defence trade-offs

Plants must balance resource allocation between growth and defence, as both processes compete for limited energy and nutrients. Activating immune responses often reduces growth rates, as defence mechanisms—such as secondary metabolite production and pathogenesis-related proteins—are energy-intensive. Hormonal cross-talk plays a central role in this trade-off, with salicylic acid (SA) and Jasmonic acid (JA) regulating immunity but antagonising growth-promoting hormones like gibberellins (Huot et al., 2014). This ensures resource conservation during pathogen attacks but results in reduced biomass and yield. Genetic and ecological factors modulate these trade-offs. Giolai and Laine (2024) observed that wild plants with extensive nucleotide-binding leucine-rich repeat receptor (NLR) repertoires exhibited reduced growth capacity, whereas domesticated crops showed less pronounced trade-offs, suggesting that breeding has partially mitigated this conflict. Emerging strategies now focus on enhancing plant resilience without compromising growth. Immunity priming, for instance, allows plants to mount a faster, stronger defence response without prolonged resource allocation to immunity. Gupta and Bar (2023) demonstrated that priming in tomatoes improved resistance while maintaining growth rates, offering promise for crop improvement. Environmental conditions further shape this trade-off. Plants in resource-rich environments prioritize growth, whereas those in pathogen-rich or nutrient-poor conditions invest more heavily in defence (Züst and Agrawal, 2017). Understanding these dynamics is crucial for optimizing growth–defence balance in agriculture to enhance both yield and resistance.

## Conclusions

Breeding for durable plant resistance remains a complex challenge due to the evolutionary adaptability of pathogens, incomplete knowledge of resistance mechanisms, and practical constraints in utilizing wild relatives. As a result, pesticides remain the primary means of crop protection despite their environmental risks and declining efficacy due to resistant pathogen strains.

While dominant *R* genes offer rapid, strong resistance, their short-lived effectiveness under high disease pressure underscores the need for quantitative disease resistance (QDR). Sustainable resistance requires an integrated approach, combining genomic advancements, conventional breeding, and multi-environment evaluations. Recent breakthroughs in genomics and high-throughput phenotyping are transforming resistance breeding, providing deeper insights into genetic diversity, host-pathogen interactions, and plant microbiomes while accelerating trait selection.

## Key recommendations for future research and application

- Prioritizing durable, broad-spectrum resistance through quantitative resistance genes (QRLs) and multi-gene pyramiding over single-gene resistance.
- Leveraging CRISPR, TALEN, and other genome-editing tools to introduce precise modifications without disrupting agronomic traits.
- Expanding genetic resources by incorporating wild germplasm, landraces, and induced mutations to enhance resistance diversity.
- Improving screening and selection techniques with high-throughput phenotyping, genomic selection, and multi-environment trials.
- Balancing growth and defence trade-offs by optimizing hormonal regulation and immunity priming for sustainable resistance without yield penalties.
- Enhancing interdisciplinary collaboration between plant pathologists, geneticists, bioinformaticians, and agronomists to integrate diverse expertise into resistance breeding.

Achieving sustainable disease resistance is not merely a breeding challenge but a global agricultural necessity. A synergistic approach, combining genomic innovations with traditional breeding, is essential to developing resilient crops capable of withstanding climate change, evolving pathogens, and global food security demands.

## Additional definitions

- Pathotypes: Pathotypes refer to subgroups or variants of a pathogenic microorganism (e.g., bacteria, fungi, or viruses) that are distinguished by their specific host range, virulence factors, or pathogenic behaviours. These differences often depend on the ability of the microorganism to infect particular host species or varieties. For instance, within a single species of pathogen, different pathotypes may show varying abilities to infect different cultivars of the same plant (Agrios, 2005). Example: Different strains of *Puccinia graminis* (stem rust pathogen) infect specific wheat cultivars, forming unique pathotypes.
- Physiological Races: Physiological races are subcategories within a pathogen species that differ in their ability to infect specific varieties or genotypes of a host. Unlike pathotypes, which may reflect broader host differences, physiological races are often identified based on specific host-pathogen interactions, typically characterized using differential hosts. This concept is widely used in plant pathology, where the classification of physiological races aids in breeding resistant crop varieties. (Van Der Plank, 1969). Example: Physiological races of *Magnaporthe oryzae* (rice blast pathogen) are identified based on their ability to infect rice varieties with different resistance genes.
- Biotrophic pathogen: A biotrophic pathogen is a type of pathogen that requires living host tissue to complete its life cycle. These pathogens establish a long-term, parasitic relationship with their host by deriving nutrients from living cells without immediately killing them. Biotrophs generally include fungus rusts (Basidiomycetes), powdery mildew pathogens (Ascomycetes), and Oomycetes (downy mildew and white rusts).
- Necrotrophic pathogen: A necrotrophic pathogen is a type of pathogen that kills host cells and tissues to derive nutrients from the dead or decaying matter. Unlike biotrophic pathogens, which rely on living host tissue, necrotrophs cause extensive damage and often produce toxins or enzymes that break down host cells, allowing them to feed on the resulting debris. Necrotrophs generally include basidiomycetes & deuteromycetes, i.e. Botrytis, Alternaria, sclerotinia, etc.
- NBS-LRR: Two types of plant NBS-LRR proteins. The two classes of NBS-LRR protein are differentiated by the N-terminal domain. TIR-NBS-LRR proteins have a Toll-interleukin-like receptor (TIR) domain, based on homology to the Drosophila Toll and mammalian Interleukin-1 (IL-1) receptors. The N-terminal region of non-TIR-NBS-LRR proteins is less defined but often contains a coiled-coil (CC) domain. In R genes, the NBS domain plays a role in intramolecular interactions with the LRR and N-terminal domains. The N-terminal domain influences the signalling pathway that will be activated upon effector recognition and may also be involved in pathogen recognition and interactions with targets of pathogen effectors region of non-TIR-NBS-LRR proteins is less defined, but often contains a coiled-coil (CC) domain.
- Pattern-Triggered Immunity (PTI) and Effector-Triggered Immunity (ETI) are two layers of plant immune defence against pathogens. PTI is the first line of defence and is activated when Pattern Recognition Receptors (PRRs) on the plant cell surface detect the pathogenic effector molecules called Pathogen-Associated Molecular Patterns (PAMPs), such as bacterial flagellin or fungal chitin. This recognition leads to a broad, immune response, which includes the production of reactive oxygen species (ROS), cell wall reinforcement, and antimicrobial compounds. PTI provides general resistance to a wide range of pathogens, but it is considered weak and can be suppressed by evolved pathogen-secreted effectors.
- ETI is considered as a specific and robust defence layer which is often activated when plant Resistance (R) proteins, often NBS-LRR proteins, recognize pathogen effectors molecules designed to surpass the PTI. ETI leads to stronger and higher impact responses, generally including the hypersensitive response (HR), which causes localized cell death to contain the pathogen.
- Hypersensitive response (HR): It is a plant defence response mechanism leading to rapid, localized programmed cell death (PCD) at the site of pathogen infection, often triggered by Effector-Triggered Immunity (ETI). Upon recognition of pathogen effectors by plant resistance (R) proteins, HR initiates a cascade of immune responses, including the production of reactive oxygen species (ROS) and activation of defence-related genes. This localized cell death restricts pathogen spread. HR is particularly more effective against biotrophic pathogens than necrotrophic pathogens.

## Common breeding methods

- The pedigree method of plant breeding involves the selection of superior individual plants from segregating populations derived from a cross between two genetically distinct parents. F_1_ plants are grown and self-pollinated to produce F_2_ progeny. Selection begins in the F_2_ generation, where desirable plants are identified based on traits of interest. These selected plants are then selfed over successive generations (F_3_, F_4_, etc.), with continued selection in each generation. A pedigree record is maintained to track the ancestry of selected plants.
- The bulk method of plant breeding involves growing a large, unselected population from a cross between two parent plants through several generations, allowing natural selection to take over generations. Initially, F_1_ seeds are grown, and subsequent generations (F_2_, F_3_, etc.) are harvested in bulk without individual plant selection. After several generations of selfing, when genetic variability has reduced, individual plants are selected from the bulk population for evaluation and breeding. This method is cost-effective and allows for natural selection to enhance traits like stress tolerance, often used in crops like wheat and barley.
- The backcross method of plant breeding involves repeatedly crossing F_1_ with one of its original parents, known as the recurrent parent, to transfer a specific trait (gene) from the donor parent into the recurrent parent’s genetic background. After each cross, progeny with the desired trait is selected and backcrossed again to the recurrent parent. This process is repeated for several generations (typically 5-6), progressively restoring the recurrent parent’s genetic makeup while retaining the targeted trait. The backcross method is widely used to incorporate disease resistance or other specific traits into elite crop varieties while maintaining their desirable characteristics.
- Recurrent selection is a plant breeding method aimed at improving populations by repeatedly selecting and inter-crossing individuals with desirable traits across multiple generations. It involves selecting superior plants from a genetically variable population and using them to create the next generation through random mating. This process enhances the frequency of favourable alleles over time while maintaining genetic diversity within the population. Recurrent selection is particularly useful for improving quantitative traits controlled by multiple genes, such as yield or drought tolerance. It is commonly applied in cross-pollinated crops like maize and forage grasses to achieve cumulative genetic gains.
- Mutation breeding involves inducing genetic mutations in plants using physical (e.g., gamma rays, X-rays) or chemical agents (e.g., EMS) to create novel genetic variation. Mutants with desirable traits, such as disease resistance, improved yield, or stress tolerance, are selected from the treated population. These mutations occur randomly, and plants with beneficial traits are identified through screening and further propagated. It is widely used to improve crops like rice, wheat, and barley, contributing to the creation of many commercially successful plant varieties.
- Marker-Assisted Backcross Breeding (MABC) combines traditional backcrossing with molecular markers to accelerate the transfer of specific genes or traits into elite plant varieties. In MABC, molecular markers linked to the desired trait (e.g., disease resistance) are used to screen progeny at each backcross generation. This allows for the precise selection of individuals carrying the target gene, while molecular markers also assist in selecting individuals with maximum genetic similarity to the recurrent parent. MABC reduces the number of backcross generations required, increases efficiency, and accelerates the development of improved varieties by integrating targeted gene introgression with molecular precision.
